# Three species of *Amphicorina* (Annelida, Sabellida, Sabellidae) from Japan, with descriptions of two new species

**DOI:** 10.3897/zookeys.187.2662

**Published:** 2012-04-27

**Authors:** Taiki Yoshihara, Shimpei F. Hiruta, Toru Katoh, Hiroshi Kajihara

**Affiliations:** 1 Laboratory of Systematics and Evolution, Department of Natural History Sciences, Hokkaido University, N10 W8, Sapporo 060-0810, Japan

**Keywords:** Taxonomy, morphology, polychaete, scanning electron microscopy, 18S rRNA, 28S rRNA

## Abstract

We describe two new species and redescribe one in the polychaete genus *Amphicorina* Claparède, 1864 (Sabellidae) from Hokkaido, Japan. *Amphicorina ascidicola* **sp. n.** differs from its 38 congeners chiefly in the reduction of the collar, but also in having three pairs of radioles, one pair of ventral radiolar appendages, a bifurcate ventral lobe on the anterior peristomial ring, six abdominal chaetigers, and a large anterior tooth on the abdominal uncini. *Amphicorina ezoensis* **sp. n.** has a crenulated collar, three pairs of radioles, and more than eight (12) abdominal chaetigers; *Amphicorina ezoensis* shares these character states with *Amphicorina anneae* (Rouse, 1994), *Amphicorina eimeri* (Langerhans, 1880), and *Amphicorina persinosa* (Ben-Eliahu, 1975), but differs from them in having two pairs of ventral radiolar appendages and a non-oblique collar. *Amphicorina mobilis* (Rouse, 1990) was previously known only from the type locality (New South Wales, Australia), but we identify our Japanese material as conspecific on the basis of morphological and molecular similarity.

## Introduction

Sabellid polychaetes in the genus *Amphicorina* Claparède, 1864 are distributed nearly worldwide; most are small (up to 6 mm in body length) and live in shallow marine environments. Since Giangrande et al.’s (1999) revision of the genus, four species have been added to *Amphicorina* by López and Tena (1999), Nogueira and Amaral (2000), and Capa and López (2004), increasing the number of species in the genus to 38. To date, however, no polychaete species has been reported from Japanese waters under the name of *Amphicorina*.

In a faunal survey around Hokkaido, northern Japan, we found three species of *Amphicorina*; we identified one as *Amphicorina mobilis* (Rouse, 1990), previously known only from Australia, whereas the other two proved to be undescribed species. Here we describe these two species as new to science and provide morphological data for *Amphicorina mobilis*; we also provide partial sequences of the 18S and 28S rRNA genes for *Amphicorina mobilis* and one of the two new species.

## Material and methods

Unless otherwise mentioned, the specimens used in this study were collected by the first author from several intertidal sites in Hokkaido, northern Japan (Akkeshi, Higashi-shizunai, Mukawa, and Muroran on the Pacific side; Okushiri-Island, Setana, and Oshoro in the Sea of Japan). For morphological observation, specimens were fixed in 10% seawater formalin and later transferred to 70% ethanol after rinsing in deionized water. For DNA extraction, most specimens were preserved in 99% ethanol, though a few living specimens were directly frozen at –10°C. Observations were made with a stereoscopic microscope, compound light microscope, and scanning electron microscope (SEM). Some intact specimens were mounted whole on glass slides, embedded in Entellan New (Merck) under a cover slip. One specimen of *Amphicorina ascidicola* sp. n. was dehydrated in an ethanol series, cleared in xylene, embedded in paraffin (m.p. 56–57°C), sectioned sagittally at 8 µm thickness, and stained using Mallory’s trichrome method (Gibson 1994). For SEM observation, specimens were dehydrated in an ethanol series, critical-point dried with CO_2_, and coated with gold. All voucher specimens have been deposited in the Hokkaido University Museum, Sapporo, Japan, catalogued under the acronym ZIHU, representing the former Zoological Institute, Hokkaido University.

DNA was extracted from either frozen or ethanol-preserved specimens using a DNeasy Tissue Kit (Qiagen, Tokyo, Japan) according to the manufacturer’s protocol. Primers used for PCR amplification of gene fragments are listed in Table 1.

Hot-start PCRs were performed using a thermal cycler (iCycler, Bio-Rad), in 10-µl reaction volumes containing 1 µl of total DNA template (approximately 10–100 ng), 1 µl Ex *Taq* buffer (TaKaRa Bio), 25 mM each dNTP, 10 µM each primer, and 2.5 U TaKaRa Ex*Taq* DNA polymerase (5 U/µl, TaKaRa Bio) in deionized water. Thermal cycling conditions were 95°C for 1 min; 35 cycles of 95°C for 30 sec, 45°C for 1.5 min, and 72°C for 3 min (18S and 28S-D3–D7) or 1 min (28S-D1); and 72°C for 7 min. PCR products were purified according to the method of Boom et al. (1990) with some modifications (Kobayashi et al. 2009). Terminator reactions were done with a BigDye Terminator v3.1 Cycle Sequencing Kit (Life Technologies) following the manufacturer’s protocol; sequencing primers are listed in Table 1. Sequencing was performed with an Applied Biosystems 3130 DNA Analyzer (Life Technologies). Base-calling and assembling were carried out using ATGC ver. 4.0.6 (GENETYX). Gene sequences were aligned and compared by using MEGA ver. 5 (Tamura et al. 2011).

**Table 1.** Primers used in this study for initial PCR amplification and/or sequencing.

	Gene fragment	Primer set	Source
**Amplification**	18S	1F (5'- TAC CTG GTT GAT CCT GCC AGT AG -3')	Giribet et al. (1996)
		9R (5'- GAT CCT TCC GCA GGT YTC ACC TAC -3')	
	28S-D1	F (5'- AAC CSC TGA AYT TAA GCA T -3')	Brown et al. (1999)
		R (5'- AAC TCT CTC MTT CAR AGT TC -3')	
	28S-D3–D7	01F (5' -GAC TAC CCC CTG AAT TTA AGC AT -3')	Kim et al. (2000)
		3KR (5'- CCA ATC CTT TTC CCG AA -3')	Hiruta (unpubl.)
**Sequencing**	18S	1F (see above)	—
		3F (5'- GTT CGA TTC CGG AGA GGG A -3')	Giribet et al. (1996)
		4R (5'- GAA TTA CCG CGG CTG CTG G -3')	
		9R (see above)	—
		18Sbi (5'- ATG GTT GCA AAG CTG AAA C -3')	Whiting et al. (1997)
		18Sa2.0 (5'- GAG TCT CGT TCG TTA TCG GA -3')	
	28S-D1	F, R (see above)	—
	28S-D3–D7	01F (see above)	—
		1KR (5'- GAC TCC TTG GTC CGY GTT TCA AG -3')	Kim et al. (2000)
		14F (5'- TGG GAC CCG AAA GAT GGT G -3')	Luan et al. (2005)
		15R (5'- CGA TTA GTC TTT CGC CCC TA -3')	Hiruta (unpubl.)
		3KR (see above)	—

## Systematics

### Genus Amphicorina

Claparède, 1864

http://species-id.net/wiki/Amphicorina

#### Type species.

*Fabricia (Amphicorina) armandi* Claparède, 1864 by monotypy.

#### Nomenclatural remarks.

The genus name was initially referred to in French as “*L’Amphicorine*” (Quatrefages 1850) for a sabellid occurring in Bréhat, France. It was later latinized as *Amphicorina* by Leuckart (1854); however, neither Quatrefages (1850) nor Leuckart (1854) combined it with any available specific name(s), and thus their usage of the name does not satisfy Article 12.2.5 of the International Code of Zoological Nomenclature (ICZN 1999). Claparède (1864) made the genus-group name available, originally as a subgenus that included only *Fabricia (Amphicorina) armandi* Claparède, 1864. Therefore, the authority of the name should be ascribed to Claparède (1864), not to Quatrefages (1850) as some authors have erroneously indicated (e.g., Nogueira and Amaral 2000; WoRMS 2010).

### Amphicorina ascidicola

sp. n.

urn:lsid:zoobank.org:act:39CC8B25-D1E9-46F4-8463-20AD2AC36E69

http://species-id.net/wiki/Amphicorina_ascidicola

Figs 1 2

#### Material examined.

**Morphology.Holotype:** ZIHU 3926, intact specimen, fixed in 10% seawater formalin, preserved in 70% ethanol, among botryllid ascidian colonies, 42°16'N, 142°27'E, Higashi-shizunai, Hokkaido, Japan, 10 June 2010. **Paratypes:** ZIHU 3927, among botryllid ascidian colonies, 42°18'N, 140°59'E, Muroran, Hokkaido, Japan, 16 April 2010; ZIHU 3928, 3929, among laminarian holdfasts, 42°33'N, 141°55'E, Mukawa, Hokkaido, Japan, 9 June 2010; ZIHU 3930, 3931, among botryllid ascidian colonies, 43°01'N, 144°50'E, Akkeshi, Hokkaido, Japan, 23 June 2009; ZIHU 3932, 3933, same data as for holotype; ZIHU 3934–3937, among laminarian holdfasts, 42°33'N, 141°55'E, Mukawa, Hokkaido, Japan, 9 June 2010 [ZIHU 3927, 3933–3937, intact specimens, fixed in 10% seawater formalin, preserved in 70% ethanol; ZIHU 3928, dissected, with half of branchial crown removed; ZIHU 3929, 3930, whole mounts on slides; ZIHU 3931, serial sagittal sections on slide; ZIHU 3932, mounted on SEM stub].

#### DNA analysis.

One specimen, among algae, 42°18'N, 140°59'E, Muroran, Hokkaido, Japan, 19 April 2011.

#### Descri

**Figure 1.**
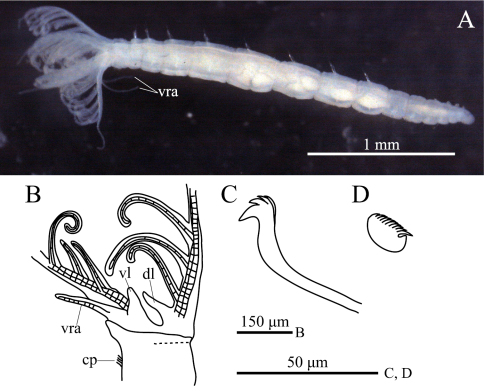
*Amphicorina ascidicola* sp. n. **A** holotype, ZIHU 3926, lateral view **B** paratype, ZIHU 3928, lateral view of inner surface of branchial crown **C** paratype, ZIHU 3930, thoracic uncinus **D** paratype, ZIHU 3931, abdominal uncinus. Abbreviations: **cp** ciliated patch **dl** dorsal lip **vl** ventral lip **vra** ventral radiolar appendage.

**Figure 2.**
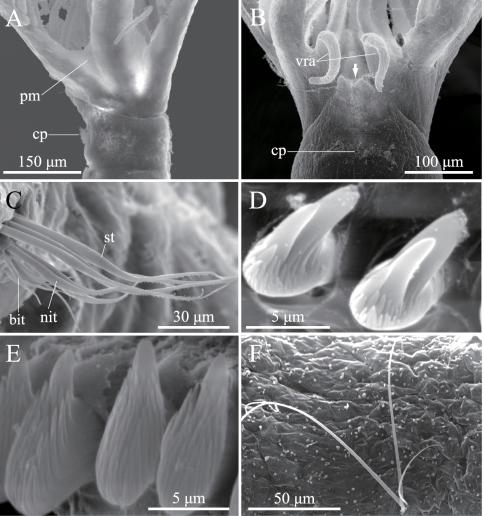
*Amphicorina ascidicola* sp. n., paratype, ZIHU 3932, SEM images. **A** basal part of radioles, lateral view **B** basal part of radioles, ventral view (arrow indicates bifurcate ventral lobe on anterior peristomial ring) **C** thoracic notochaetae on the 5th chaetiger **D** thoracic uncini **E** abdominal uncini **F** abdominal chaetae. Abbreviations: **bit** bayonet inferior thoracic notochaetae **cp** ciliated patch **nit** narrowly hooded inferior thoracic notochaetae **pm** palmate membrane **st** superior thoracic notochaetae **vra** ventral radiolar appendage.

**ption.** Body with eight thoracic and six abdominal chaetigers (Fig. 1A). Total length 2.8 mm, crown length 0.7 mm, maximum body width 0.3 mm. Three pairs of radioles, with lateral flanges; proximal 1/7 of radioles connected by palmate membrane; each radiole with six pairs of pinnules ending with terminal pinnule; all pinnules ending at same height as terminal pinnule (Fig. 1A). Each radiole with two longitudinal internal cellular supporting axes; each pinnule with one internal cellular supporting axis. One pair of ventral radiolar appendages present, with one internal cellular supporting axis, nearly 1/2 radiole length (Fig. 1A). One pair of elongate dorsal lips present, with neither pinnular nor radiolar appendages; one pair of triangular ventral lips present (Fig. 1B). Distal end of ventral lobe on anterior peristomial ring bifurcate (Fig. 2B). Posterior peristomial ring collar absent; border between anterior and posterior peristomial ring obscure (Figs 1B, 2A, 2B). Small ciliated patch on posterior peristomial ring (Figs 1B, 2A, 2B). One pair of red eyes present on peristomium (not visible in preserved specimens). Glandular ridge absent.

Superior thoracic notochaetae elongate, narrowly hooded, 3–5 per fascicle (Fig. 2C). Inferior thoracic notochaetae bayonet type, five per fascicle in first thoracic chaetiger; second to eighth thoracic chaetigers with 3–4 narrowly hooded and 5 bayonet-type inferior thoracic notochaetae (Fig. 2C). Thoracic acicular uncini 5–7 per torus; each uncinus with three rows of irregular-sized teeth above main fang (Figs 1C, 2D). Abdominal uncini quadrangular, with eight rows of teeth above large basal tooth (Figs 1D, 2E), 5–15 uncini per torus. Abdominal neurochaetae needle-like capillaries in form, three per fascicle (Fig. 2F).

Pygidium rounded, with one pair of red eyes; color of eyes faded in preserved specimens.

One pair of statocysts in first thoracic chaetiger evident in living state. Oocytes found in sixth to eighth thoracic chaetigers.

#### DNA analysis.

We obtained sequences for two of the three target gene fragments for this species (GenBank accession numbers AB646764, 18S, 1677 bp; AB646765, 28S-D1, 377 bp); we were unable to sequence 28S-D3–D7. Both strands were sequenced for 28S-D1; part of the 18S sequence is based on only one strand. Among species of *Amphicorina*, DNA sequence data were available only for *Amphicorina mobilis* (Rousset et al. 2004; Kupriyanova and Rouse 2008). In a 1687 bp alignment of 18S sequences, *Amphicorina ascidicola* differed in sequence from the Australian (GenBank accession number EF116206, Kupriyanova and Rouse 2008) and Japanese specimens (AB646764) of *Amphicorina mobilis* by 15 indels and 17 substitutions in each case. In a 321 bp alignment of the 28S-D1 region, *Amphicorina ascidicola* differed in sequence fromthe Australian (EF116217, Kupriyanova and Rouse 2008) and Japanese (AB646765) specimens of *Amphicorina mobilis* by five substitutions and one indel in each case.

#### Etymology.

The specific name, a noun, is a combination of *ascidia* (sea squirt) and -*cola* (dweller), referring to the fact that the species was frequently found among botryllid ascidian colonies.

#### Remarks.

Among the 38 congeners, the following eight species have been reported to exhibit a reduction in the collar [= absence of posterior peristomial ring collar] as in *Amphicorina ascidicola*: *Amphicorina alata* (Ehlers, 1897), *Amphicorina brevicollaris* (Rouse, 1990), *Amphicorina gracilis* (Hartman, 1969), *Amphicorina grahamensis* Giangrande, Montanaro & Castelli,^ 1999^, *Amphicorina minuta* (Berkeley & Berkeley, 1932), *Amphicorina neglecta* (Banse, 1957), *Amphicorina pectinata* (Banse, 1957), and *Amphicorina triangulata* López & Tena, 1999. However, the present new species can be distinguished from those by the combination of characters and their states summarized in Table 2.

**Table 2.** Comparison of characters in species of *Amphicorina* with a reduced collar.

Taxa	Characters					Source
	Number of pairs of radioles	Number of pairs of ventral radiolar appendages	Ventral lobe on anterior peristomial ring	Number of abdominal chaetigers	Inferior tooth of abdominal uncini	
*Amphicorina alata* (Ehlers, 1897)	4	1	absent	6	small	Ehlers (1897), Giangrande et al. (1999), López and Tena (1999)
*Amphicorina brevicollaris* (Rouse, 1990)	4	2	bifurcate	7	small	Rouse (1990), Giangrande et al. (1999), López and Tena (1999)
*Amphicorina gracilis* (Hartman, 1969)	3	0	absent	8	small	Hartman (1969), Giangrande et al. (1999), López and Tena (1999)
*Amphicorina grahamensis* Giangrande, Montanaro & Castelli, 1999	3	1	absent	5	large	Giangrande et al. (1999), López and Tena (1999)
*Amphicorina minuta* (Berkeley & Berkeley, 1932)	2	?	absent	5	large	Berkeley and Berkeley (1932), Giangrande et al. (1999), López and Tena (1999)
*Amphicorina neglecta* (Banse, 1957)	4	1	absent	6	large	Banse (1957), Giangrande et al. (1999), López and Tena (1999)
*Amphicorina pectinata* (Banse, 1957)	4	2	bifurcate	6	large	Banse (1957), Giangrande et al. (1999), López and Tena (1999)
*Amphicorina triangulata* López & Tena, 1999	4	1	bifurcate	6	large	López and Tena (1999)
*Amphicorina ascidicola* sp. n	3	1	bifurcate	6	large	Present study

### Amphicorina ezoensis sp. n.

urn:lsid:zoobank.org:act:3B810496-A96D-4D12-904F-B3C695EA48FD

http://species-id.net/wiki/Amphicorina_ezoensis

Figs 3 4

#### Material examined.

**Holotype:** ZIHU 4255, fixed in 10% seawater formalin, preserved in 70% ethanol, among algae, 42°33'N, 139°50'E, Setana, Hokkaido, Japan, 10 May 2010. **Paratypes:** ZIHU 4254, mounted on SEM stub, same data as for holotype; ZIHU 4270, fixed in 10% seawater formalin, preserved in 70% ethanol, same data as for holotype.

#### Description.

**Figure 3:**
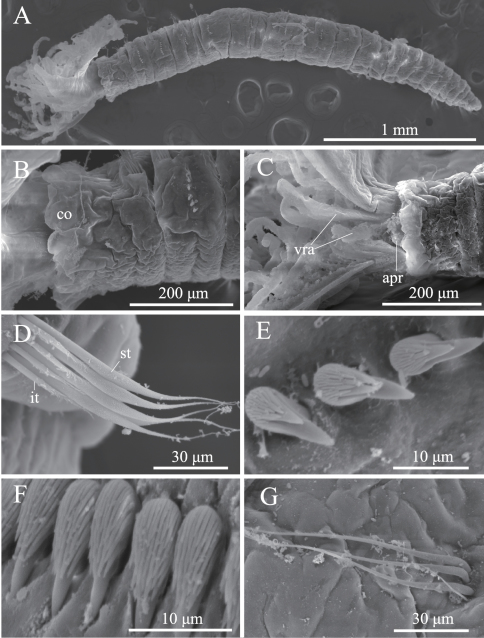
**.** *Amphicorina ezoensis* sp. n., paratype, ZIHU 4254, SEM images. **A** entire worm, lateral view **B** collar and first thoracic segment, lateral view **C** basal part of radioles, ventral view **D** thoracic notochaetae on the 4th chaetiger **E** thoracic uncini **F** abdominal uncini **G** abdominal chaetae. Abbreviations: **apr** anterior peristomial ring **co** collar **it** inferior thoracic notochaetae **st** superior thoracic notochaetae **vra** ventral radiolar appendage.

**Figure 4.**
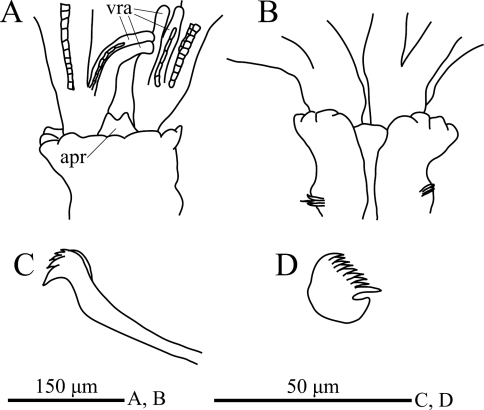
*Amphicorina ezoensis* sp. n., holotype, ZIHU 4255. **A** collar, ventral view **B** collar, dorsal view **C** thoracic uncinus **D** abdominal uncinus. Abbreviations: **apr** anterior peristomial ring **vra** ventral radiolar appendage.

Eight thoracic and 12 abdominal chaetigers (Fig. 3A). Total length 3.1 mm, crown length 0.6 mm, maximum body width 0.3 mm. Three pairs of radioles, with lateral flanges; proximal 1/2 of radioles connected by palmate membrane. Each radiole with two longitudinal internal cellular supporting axes; each pinnule with one internal cellular supporting axis. Ventral-most radiole with two appendages on each side (Fig. 4A); these appendages (arranged dorsally and ventrally) being almost 1/2 radiole length, and only dorsal one having one internal cellular supporting axis. Distal end of ventral lobe on anterior peristomial ring bifurcate, extending slightly beyond collar margin (Figs 3C, 4A). Posterior peristomial ring collar crenulate (Figs 3A, 3B, 3C, 4A, 4B), with dorsal gap (Fig. 4B). Ciliated patch absent on posterior peristomail ring (Figs 3C, 4A). Glandular ridge on second chaetiger present.

**Figure 5.**
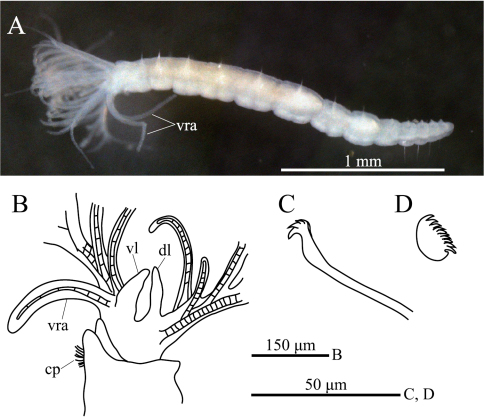
*Amphicorina mobilis* (Rouse, 1990). **A** ZIHU 3938, entire worm, lateral view **B** ZIHU 3939, lateral view of inner surface of branchial crown **C** ZIHU 3940, thoracic uncinus **D** ZIHU 3941, abdominal uncinus. Abbreviations: **cp** ciliated patch **dl** dorsal lip **vl** ventral lip **vra** ventral radiolar appendage.

**Figure 6.**
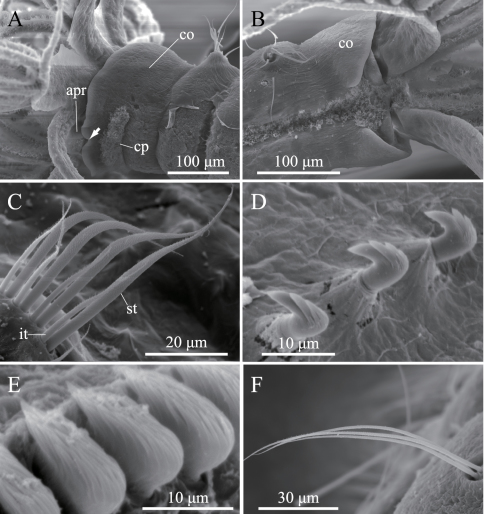
*Amphicorina mobilis* (Rouse, 1990), ZIHU 3942, SEM images. **A** collar segment, ventrolateral view (arrow indicates ventral notch of collar) **B** collar segment, dorsal view **C** thoracic notochaetae on the 5th chaetiger **D** thoracic uncini **E** abdominal uncini **F** abdominal chaetae. Abbreviations: **apr** anterior peristomial ring **co** collar **cp** ciliated patch **it** inferior thoracic notochaeta **st** superior thoracic notochaetae**.**

Superior thoracic notochaetae elongate, narrowly hooded, 4–5 per fascicle (Fig. 3D). Inferior thoracic notochaetae bayonet type, four per fascicle; no elongate, narrowly hooded chaetae (Fig. 3D). Thoracic acicular uncini 4–5 per torus; each uncinus having four rows of teeth above main fang (Figs 3E, 4C). Abdominal uncini quadrangular, with eight rows of teeth above large basal tooth (Figs 3F, 4D), 2–9 uncini per fascicle; number of uncini decreasing posteriorly, with eight uncini on first and second abdominal chaetigers, nine on third. Abdominal neurochaetae 2–4 per fascicle, needle-like capillaries in form (Fig. 3G). Pygidium rounded. Peristomial and pygidial eyes and statocysts not visible in preserved specimens. Oocytes found in fourth and fifth thoracic chaetigers.

#### Etymology.

The specific epithet is an adjective derived from *Ezo*, the old place name for Hokkaido, in combination with the Latin suffix -*ensis*.

#### Remarks.

*Amphicorina ezoensis* is similar to *Amphicorina anneae* (Rouse, 1994), *Amphicorina eimeri* (Langerhans, 1880), and *Amphicorina persinosa* (Ben-Eliahu, 1975) in having a crenulate collar, three pairs of radioles, and more than eight abdominal chaetigers. *Amphicorina ezoensis* differs from *Amphicorina persinosa* in the shape of the collar. In *Amphicorina ezoensis*, the anterior edge of the collar is perpendicular to the anterior-posterior body axis, and the collar completely covers the anterior peristomium so that the latter is not visible laterally, while in *Amphicorina persinosa* the collar is oblique in lateral view so that the anterior peristomium is visible, although the angle of the collar is often determined by how the specimen was fixed. *Amphicorina ezoensis* further differs from *Amphicorina anneae* and *Amphicorina persinosa* in the number of ventral radiolar appendage(s). *Amphicorina ezoensis* has two pairs of appendages, while *Amphicorina anneae* and *Amphicorina persinosa* have one pair. The number of ventral radiolar appendage(s) was not mentioned in the original description of *Amphicorina eimeri* (Langerhans, 1880), but Banse (1957: 72) noted “ventral wenigstens ein Filament” (ventrally at least one filament); Giangrande et al. (1999: 197, Table 1) indicated the species has one pair of appendages, while Rouse (1990: Table 1) lists “1?”. *Amphicorina ezoensis* also differs from *Amphicorina eimeri* in that the former possesses elongate, narrowly hooded thoracic chaetae, while the latter has broadly hooded thoracic chaetae (Rouse 1990, Giangrande et al. 1999). *Amphicorina ezoensis* also differs from *Amphicorina eimeri* in the number of the abdominal chaetigers (12 vs. 10).

We were unable to obtain DNA sequence data for this species due to the paucity of material.

### Amphicorina mobilis

(Rouse, 1990)

http://species-id.net/wiki/Amphicorina_mobilis

Figs 5 6

Oriopsis mobilis Rouse, 1990: 230–231, fig. 5a–i.
Amphicorina mobilis : Giangrande et al. 1999: 197, Table 1; Nogueira and Amaral 2000: 622; Rousset et al. 2004: Table 3; 2007: 47, Table 1; Kupriyanova and Rouse 2008: 1177, Table 1; Capa et al. 2010: 2, Table 1; Huang et al. 2011: 3, Table 1.
Amphicorina ? sp. Giangrande et al. 1999: 199–200, fig. 4a–g.
Fabricia ventrilinguata ?: Fitzhugh 1990: 14. Not Johansson (1922).
Fabricia sabella ?: Imajima and Hartman 1964: 366. Not Ehrenberg (1836).

#### Material examined.

**Morphology.**Twenty-five specimens. ZIHU 3938, among botryllid ascidian colonies, 42°16'N, 142°27'E, Higashi-shizunai, Hokkaido, Japan, 10 June 2010; ZIHU3939, among algae, 42°16'N, 142°27'E, Higashi-shizunai, Hokkaido, Japan, 10 June 2010; ZIHU 3940, 3941, among algae, 42°06'N, 139°25'E, Okushiri-Island, Hokkaido, Japan, 9 May 2010; ZIHU 3942, among algae, 42°16'N, 142°27'E, Higashi-shizunai, Hokkaido, Japan, 10 June 2010; ZIHU 3943, among botryllid ascidian colonies, 43°12'N, 140°51'E, Oshoro, Hokkaido, Japan, 16 October 2009; ZIHU 3944, among algae, 42°16'N, 142°27'E, Higashi-shizunai, Hokkaido, Japan, 10 June 2010; ZIHU 3945, among *Mytilus*, 43°12'N, 140°51'E, Oshoro, Hokkaido, Japan, 23 May 2010; ZIHU 3946, among algae, 43°12'N, 140°51'E, Oshoro, Hokkaido, Japan, 24 May 2010; ZIHU 3947, among algae, 42°06'N, 139°25'E, Okushiri-Island, Hokkaido, Japan, 9 May 2010; ZIHU 3948, among algae, 43°12'N, 140°51'E, Oshoro, Hokkaido, Japan, 23 March 2010; ZIHU 4273, two specimens, among sessile organisms on culture panel for the vase tunicate *Ciona intestinalis* (Linnaeus, 1767) hung from a raft, 35°09'N, 139°36'E, Misaki, Kanagawa, Japan, 22 February 2012, K. Kakui leg; ZIHU 4274, three specimens, same locality data as ZIHU 4273 [ZIHU 3938, 3043–3948, 4273, intact specimens, fixed in 10% seawater formalin, preserved in 70% ethanol; ZIHU3939, dissected, with half of the branchial crown removed; ZIHU 3940, 3941, whole mount on slide; ZIHU 3942, mounted on SEM stub; ZIHU 4273, fixed in Bouin's fluid, preserved in 70% EtOH; ZIHU 4274, fixed and preserved in 99% EtOH].

#### DNA analysis.

Two specimens: one collected among algae, 42°06'N, 139°25'E, Okushiri-Island, Hokkaido, Japan, 9 May 2010; the other collected among laminarian holdfasts, 42°18'N, 140°59'E, Muroran, Hokkaido, Japan, 19 April 2011.

#### Description.

Complete specimens have eight thoracic and five abdominal chaetigers (Fig. 5A). Total length 1.2–3.2 mm (mean, 2.3 mm; n = 9), crown length 0.2–0.6 mm (mean, 0.4 mm; n = 9), maximum width 0.3 mm. Three pairs of radioles with lateral flanges; proximal 1/7 of radioles connected by palmate membrane; each radiole with six pairs of pinnules ending with terminal pinnule; all pinnules ending at same height as terminal pinnule. Each radiole with two longitudinal internal cellular supporting axes; each pinnule with one internal cellular supporting axis. One pair of ventral radiolar appendages present, nearly as long as radioles, with one internal cellular supporting axis (Fig. 5B). One pair of elongate dorsal lips present, with neither pinnular nor radiolar appendages; one pair of triangular ventral lips present (Fig. 5B). Distal end of ventral lobe on anterior peristomial ring bifurcate, extending slightly beyond collar margin (Figs 5B, 6A). Posterior peristomial ring collar margin smooth, with small ventral notch (Fig. 6A). Collar with dorsal gap (Fig. 6B). Small ciliated patch located on posterior peristomial ring (Figs 5B, 6A). One pair of red eyes present on peristomium (not visible in preserved specimens). Glandular ridge on second chaetiger (not visible in preserved specimens).

Superior thoracic notochaetae elongate, narrowly hooded, 3–7 per fascicle (n = 10; usually 4–5 within single specimen) (Fig. 6C). Inferior thoracic notochaetae bayonet type, 3–7 per fascicle (n = 10) (Fig. 6C). Thoracic acicular uncini 3–8 per torus (n = 10); each uncinus with three rows of teeth above main fang; teeth on first row distinctly larger than those on upper rows (Figs 5C, 6D). Abdominal uncini quadrangular, with nine rows of teeth above small basal tooth (Figs 5D, 6E), 4–17 uncini per fascicle (n = 10). Abdominal neurochaetae three in number (two in the smallest specimen, ZIHU 3947) (n = 10), needle-like capillaries in form (Fig. 6F).

Pygidium rounded, with one pair of red eyes; color of eyes faded in preserved specimens.

In living specimens, paired statocysts are evident in first thoracic chaetiger; oocytes found in sixth to eighth thoracic chaetigers.

#### DNA analysis.

We obtained sequences for each of the three target gene fragments for this species (GenBank accession numbers AB646767, 18S, 1777 bp; AB646763, 28S-D1, 380 bp; AB646766, 28S-D3–7, 1998 bp). Both strands were sequenced for 18S and 28S-D1; part of the 28S-D3–7 sequence is based on only one strand. In a reliably aligned 320 bp stretch of the 28S-D1 sequence, we observed one indel difference (gap) from the aligned homologous sequence from an Australian specimen (EF116217, Kupriyanova and Rouse 2008). In an aligned 1779-bp region of 18S, we observed two indel differences between our sequence and that from an Australian specimen (EF116206, Kupriyanova and Rouse 2008).

#### Remarks.

*Amphicorina mobilis* was previously known only from Australia (Rouse 1990). A similar form was reported by Giangrande et al. (1999) as *Amphicorina* sp. from the Mediterranean, but it was not identified to species due to the poor condition of the specimens available.

Our specimens are quite similar to those in the original description of *Amphicorina mobilis* by Rouse (1990), with differences in body size, in ranges of number of chaetae and pinnules, and in the arrangement of teeth in the thoracic uncini. The Australian specimens were reported to be 1.1 mm in body length, while specimens from this study are up to 3.2 mm. Numbers of chaetae and pinnules reported by Rouse (1990), followed by those in our Japanese material in parentheses, are: thoracic superior notochaetae 3–4 (3–7), thoracic inferior notochaetae 3–4 (3–7), thoracic uncini 3–5 (3–8), abdominal uncini 3–9 (4–17), and abdominal neurochaetae 1–2 (2–3); and pairs of pinnules 5 (6). Rouse (1990) reported that *Amphicorina mobilis* has thoracic unicini with two rows of teeth above the main fang; the first row above the main fang has a large central tooth flanked by smaller teeth. By comparison, our specimens possessed no smaller teeth juxtaposing the large central tooth above main fang.

The DNA sequences shed little light on species identity, as the 18S and 28S genes evolve too slowly to reliably detect significant variation between closely related species, and the few mutations detected could as well be attributed to PCR or sequencing errors. Nonetheless, the Australian and (putative) Japanese populations of *Amphicorina mobilis* showed much less sequence divergence from one another than did either from a clearly morphologically distinct species, *Amphicorina ascidicola* sp. n. which lends weight to the interpretation that the Japanese and Australian populations are conspecific.

We consider that the specimen now labeled as the holotype of *Fabricia ventrilinguata* Johansson, 1922 deposited in the Zoologiska Museet, Uppsala (ZUM 206), might represent *Amphicorina mobilis*. We concur with Fitzhugh (1990) in that the original specimen (i.e., true *Fabricia ventrilinguata*), or its label, was likely to be replaced later by accident. *Fabricia ventrilinguata* was originally described from Misaki, Japan, based on a polychaete collection made by Sixten Bock in 1914. Imajima and Hartman (1964) and Fitzhugh (1990) observed the “holotype” of *Fabricia ventrilinguata* and pointed out discrepancies between Johansson’s (1922) original description and the actual specimen; these include (character states in the parentheses refer to those given in Johansson (1922) vs. those in the actual specimen): the length of the body (6.5 mm vs. 2.1 mm), the number of thoracic chaetigers (4 vs. 8), and the posterior peristomial ring collar (absent vs. present). Because Johansson’s (1922) original description lacks important morphological characters used in identifying genera and species within Fabriciidae, the name *Fabricia ventrilinguata* should be treated as a *nomen dubium*. On the other hand, specimen ZUM 206 redescribed by Fitzhugh (1990) is applicable to *Amphicorina*, and possibly to *Amphicorina mobilis*. Taking into account that ZUM 206 might represent an undescribed species, however, further examination for a positive identification is necessary with respect to the shape of the thoracic notochaetae and the size of the tooth on the abdominal uncini. The fact that our specimens from Misaki, the same locality as ZUM 206, were identified as *Amphicorina mobilis* with certainty does not contradict our speculation that ZUM 206 would actually represent *Amphicorina mobilis*. If this is the case, *Amphicorina mobilis* was present in Misaki before 1914.

#### Distribution.

Southeastern Australia and eastern Japan; questionably the Mediterranean. At present we have no definitive evidence whether this distribution pattern represents a natural one or has been artificially expanded. If the latter is the case, much more thorough population genetic studies may reveal the native locality and invasion pathways. Incidentally, among sabellids, *Sabella spallanzanii* (Gmelin, 1791) has been reported to be introduced from European waters to Australia, possibly either via ballast water or hull fouling (Patti and Gambi 2001). The same species has been also reported from New Zealand, introduced either via Australia or directly from Europe (Read et al. 2011). Another sabellid, *Branchiomma bairdi* (McIntosh, 1885), originally distributed in the Caribbean Sea, was recorded in the southern Gulf of California; hull fouling was considered the most probable vector for the translocation (Tovar-Hernández et al. 2009).

## Supplementary Material

XML Treatment for Amphicorina

XML Treatment for Amphicorina ascidicola

XML Treatment for Amphicorina ezoensis

XML Treatment for Amphicorina mobilis
